# Editorial: Advancing radioprotection: mechanisms, interventions, and therapeutic strategies

**DOI:** 10.3389/fphar.2026.1942789

**Published:** 2026-08-14

**Authors:** Wenfeng Gou, Hongying Yang, Wei Li

**Affiliations:** 1 Institute of Radiation Medicine, Chinese Academy of Medical Sciences & Peking Union Medical College, Tianjin, China; 2 State Key Laboratory of Radiation Medicine and Protection, School of Radiation Medicine and Protection, Suzhou Medical College of Soochow University, Suzhou, Jiangsu, China; 3 Faculty of Pharmaceutical Sciences, Toho University, Funabashi, Japan

**Keywords:** drug development, FLASH radiotherapy, glutamine, immune modulation, radiation injury, radioprotection, tardigrade

Ionizing radiation has become indispensable in modern medicine, energy production, and space exploration; however, its deleterious effects on normal tissues remain a formidable challenge. Despite decades of research, amifostine remains the sole FDA-approved radioprotectant, underscoring an urgent and unmet need for more effective, safer, and mechanistically diverse countermeasures against radiation-induced injury (Kamran et al., 2016; King et al., 2020). Therefore, an in-depth understanding of the molecular mechanisms underlying radiation injury and the interactions among various cellular and molecular mediators holds great promise for the development of novel radioprotective drugs and treatment strategies. In the current Research Topic, “Advancing Radioprotection: Mechanisms, Interventions, and Therapeutic Strategies”, we aimed to collect the most recent progress made in radioprotection research. In total, after being peer-reviewed, five manuscripts, composed of three review articles, one original research article, and one case report authored by 31 researchers worldwide, were successfully accepted for publication. These contributions span immune regulation, nutrient-based protection, bioinspired drug discovery, and advanced physical modalities, offering a multifaceted perspective on the future of radioprotection.

Radiation injury is fundamentally an immunopathological process. Wu et al. provide a comprehensive review of how immune cells and cytokines orchestrate both the inflammatory cascade and subsequent repair process following irradiation. They systematically dissect the dual roles of macrophages (M1/M2 polarization), T helper cell subsets (Th1, Th2, Th17, and Tregs), neutrophils, and dendritic cells, while also mapping the cytokine networks—including IL-1 family members, TNF-α, TGF-β1, IL-6, IL-17, and interferons—that govern tissue outcomes. Their work highlights that the temporal dynamics and context-dependent functions of these immune mediators determine whether inflammation resolves or progresses to fibrosis, identifying TREM-1 on macrophages, the IL-33/ST2 axis, and Treg modulation as promising therapeutic nodes. This review establishes a crucial framework for immune-based radioprotective strategies and biomarker development.

Building on mechanistic insights, Lu et al. investigate a targeted nutritional intervention for radiation-induced intestinal injury, a common dose-limiting toxicity in pelvic radiotherapy. Using a rat abdominal irradiation model and HT-29 cell cultures, they demonstrate that glutamine pre-administration protects Lgr5^+^ intestinal stem cells and promotes goblet cell differentiation via the mTOR/Notch1 signaling axis. Notably, glutamine suppressed radiation-induced Notch1 overactivation while activating mTOR and its downstream effectors S6K1 and 4E-BP1. Pharmacological interventions with the mTOR inhibitor rapamycin and the Notch1 agonist Jagged-1 confirmed that mTOR acts upstream to modulate Notch1, thereby governing mucosal barrier integrity. This work provides compelling preclinical evidence that glutamine, a safe, affordable nutrient, could serve as a proactive radioprotective agent for gastrointestinal protection, consistent with previous findings on glutamine’s protective effects against radiation-induced intestinal injury (Giris et al., 2006).

Perhaps the most conceptually transformative contribution comes from Cui et al., who review the translational pharmacology of tardigrade-derived radioprotectants. Tardigrades routinely survive doses exceeding 5 kGy of ionizing radiation, three orders of magnitude beyond human lethal thresholds. Their remarkable tolerance relies on a sophisticated defense repertoire: the genome-shielding protein Dsup, intrinsically disordered proteins such as CAHS and MAHS that vitrify the cytoplasm, antioxidant pigments acquired through horizontal gene transfer, and hyper-efficient DNA repair networks (Li et al., 2024). The authors map a development pipeline from recombinant proteins and mRNA therapeutics to peptidomimetics and biomimetic nanomaterials, while candidly addressing hurdles of immunogenicity, scalable bioprocessing, and targeted delivery. This review transforms an evolutionary curiosity into a tangible roadmap for next-generation radioprotectants suitable for radiotherapy, nuclear emergencies, and deep-space missions.

Complementing biological approaches, Zhi et al. examine FLASH radiotherapy, a physical modality that delivers ultra-high dose rates exceeding 40 Gy/s to achieve tumor control while sparing normal tissues, a phenomenon known as the “FLASH effect”. Given the testes’ exquisite radiosensitivity and the critical importance of fertility preservation, the authors systematically review FLASH effects across rodent organs, including skin, brain, heart, lung, intestine, and spleen. They also analyze the dose-rate-dependent responses of spermatogonia, Sertoli cells, and Leydig cells. Although direct evidence for the FLASH effect in testicular tissue remains lacking, the authors put forward several mechanistic hypotheses that warrant targeted investigation, such as oxygen depletion, reduced ROS generation, attenuated DNA damage, and immunomodulation. This review underscores the pressing need for dedicated studies of testicular FLASH responses to inform fertility-sparing radiotherapy protocols.

Finally, Zhang et al. contribute a clinically instructive case report. A 57-year-old male with radiation-induced diffuse hemorrhagic gastroduodenitis, refractory to endoscopic intervention and arterial embolization, achieved sustained hemostasis with oral thalidomide. While thalidomide is known for its anti-angiogenic and immunomodulatory properties, its efficacy in this context highlights the potential for repurposing existing drugs with established safety profiles. This represents a pragmatic bridge between mechanistic research and bedside management.

In conclusion, an increasing number of radioprotective strategies have attracted attention in recent years. Bioactive natural compounds, including polysaccharides, polyphenols, saponins, alkaloids, and peptides, have emerged as key research targets for next-generation radioprotective drugs due to their low toxicity and multi-target synergistic effects (Li et al., 2025). Each approach has unique advantages and limitations, and fully utilizing their strengths while circumventing their weaknesses is of great importance for the clinical management of radiation injury. Collectively, these five articles embody the paradigm shift from a “one-size-fits-all” approach to a stratified, mechanism-driven radioprotection strategy. They span the therapeutic spectrum: from immune modulation and nutrient-based protection to bioinspired proteins, advanced physical delivery, and drug repurposing. We extend our sincere gratitude to all authors for their insightful contributions, to the reviewers for their rigorous evaluations, and to the Frontiers editorial team for their unwavering support. We trust this Research Topic will serve as a valuable reference for researchers and clinicians striving to advance radioprotection from bench to bedside.
